# The causal relationship between air pollution, obesity, and COVID-19 risk: a large-scale genetic correlation study

**DOI:** 10.3389/fendo.2023.1221442

**Published:** 2023-10-05

**Authors:** Jingwei Zhang, Jie Wen, Xin Wan, Peng Luo

**Affiliations:** ^1^ Department of Neurosurgery, Xiangya Hospital, Central South University, Changsha, China; ^2^ Hypothalamic Pituitary Research Centre, Xiangya Hospital, Central South University, Changsha, China; ^3^ National Clinical Research Center for Geriatric Disorders, Xiangya Hospital, Central South University, Changsha, China; ^4^ Department of Oncology, Zhujiang Hospital, Southern Medical University, Guangzhou, China

**Keywords:** COVID-19, air pollution, obesity, Mendelian randomization, mediation

## Abstract

**Objective:**

Observational evidence reported that air pollution is a significant risk element for numerous health problems, such as obesity and coronavirus disease 2019 (COVID-19), but their causal relationship is currently unknown. Our objective was to probe the causal relationship between air pollution, obesity, and COVID-19 and to explore whether obesity mediates this association.

**Methods:**

We obtained instrumental variables strongly correlated to air pollutants [PM2.5, nitrogen dioxide (NO_2_) and nitrogen oxides (NOx)], 9 obesity-related traits (abdominal subcutaneous adipose tissue volume, waist-to-hip ratio, body mass index, hip circumference, waist circumference, obesity class 1-3, visceral adipose tissue volume), and COVID-19 phenotypes (susceptibility, hospitalization, severity) from public genome-wide association studies. We used clinical and genetic data from different public biological databases and performed analysis by two-sample and two-step Mendelian randomization.

**Results:**

PM2.5 genetically correlated with 5 obesity-related traits, which obesity class 1 was most affected (beta = 0.38, 95% CI = 0.11 - 0.65, *p* = 6.31E-3). NO_2_ genetically correlated with 3 obesity-related traits, which obesity class 1 was also most affected (beta = 0.33, 95% CI = 0.055 - 0.61, *p* = 1.90E-2). NOx genetically correlated with 7 obesity-related traits, which obesity class 3 was most affected (beta = 1.16, 95% CI = 0.42-1.90, *p* = 2.10E-3). Almost all the obesity-related traits genetically increased the risks for COVID-19 phenotypes. Among them, body mass index, waist circumference, hip circumference, waist-to-hip ratio, and obesity class 1 and 2 mediated the effects of air pollutants on COVID-19 risks (*p* < 0.05). However, no direct causal relationship was observed between air pollution and COVID-19.

**Conclusion:**

Our study suggested that exposure to heavy air pollutants causally increased risks for obesity. Besides, obesity causally increased the risks for COVID-19 phenotypes. Attention needs to be paid to weight status for the population who suffer from heavy air pollution, as they are more likely to be susceptible and vulnerable to COVID-19.

## Introduction

First reported in late 2019, coronavirus disease 2019 (COVID-19), caused by severe acute respiratory syndrome coronavirus 2 (SARS-CoV-2), is a pandemic affecting people’s health worldwide (1). The latest epidemiological data from World Health Organization shows that COVID-19 has caused nearly 757 million infections and more than 6.85 million deaths worldwide as of Feb. 23, 2023. Over 40% of COVID-19 survivors suffered from unresolved symptoms at four months, regardless of hospitalization status (2). Over 10% of people survived with long-term impacts on multiple organ systems, known as long COVID-19 (3). Although vaccination has reduced the incidence of severe COVID-19 to some extent, no specific treatment can target SARS-CoV-2 infection until now other than hormonal drug therapy for oxygen-dependent COVID-19 patients (4). Many exposures increase the susceptibility and severity of COVID-19, such as cardiovascular and metabolic disorders, high BMI, C-reactive protein (CRP), and smoking (5). Numerous genome-wide association studies (GWASs) in healthy populations of patients have allowed us to begin identifying the genetic correlation between exposure and disease at the genetic level. Identifying and uncovering novel factors influencing COVID-19 is essential for understanding this pandemic and enhancing its treatment.

With the rapid development of socioeconomic, air pollution remains a global health threat. Air pollution contributes to many acute and chronic diseases, such as respiratory tumors, pneumonia, chronic obstructive pulmonary disease (COPD), stroke, and heart and mental health disease (6, 7). Air pollution, including particulate matter with a diameter smaller than 2.5 µm (PM2.5), nitrogen oxides (NOx), nitrogen dioxides (NO_2_), and ozone (O_3_), are common and highly concentrated substances in modern cities and are relevant to people’s daily lives (8). Air pollution molecules entering the respiratory tract can cause respiratory tract damage through pathological mechanisms such as inflammation and oxidative stress, thereby increasing the susceptibility and severity of respiratory diseases (9). Recent observational research indicates that PM2.5 and carbon monoxide can increase the number of daily cases, cumulative cases, and cumulative deaths of COVID-19 (10). However, the causal relationship between these components (PM2.5, NOx, and NO_2_) and COVID-19 risk (susceptibility, hospitalization, and severity) remains largely unclear.

Due to multiple factors (genetics, epigenetics, environment, socioeconomic status, etc.), obesity has become another health problem that plagues a large number of young people. It is a medical problem that increases the risk for certain illnesses, such as cardiovascular disease, metabolic disease, neurodegenerative disease, and certain tumors (11, 12). Obesity generally cannot often be prevented through just eating a healthier diet, increasing activity, and behavioral change as evidenced by the fact that most obesity prevention strategies geared towards healthy diets, increasing activity, and other behavioral changes have been ineffective or at best only minimally effective (13). Obesity often plays a very important role as a mediator in the influence of many environmental factors on various diseases. In particular, BMI, an important obesity-related trait, directly contributed to COVID-19 Susceptibility (14).

Genome-wide association study (GWAS) is a genetics research methodology used to identify genomic variants that are statistically associated with the risk of a disease or a specific trait. However, the relationship between other obesity traits and COVID-19 still needs to be further investigated, which is made possible by the increasing availability of GWAS data related to these traits. Moreover, studies suggest that prolonged environmental exposure is strongly associated with obesity and/or metabolic disease. A multicenter study found that positive association between chronic exposure to PM2.5 during working and fasting plasma glucose among asymptomatic adults (15). A meta-analysis approach indicated that PM2.5 increase obesity (OR = 1.96, 95% CI = 1.21-3.18) among adolescents in Latin American cities (16). Considering the close connection between air pollution, obesity, and COVID-19, it is vital to explore their causal relationship and mediating role based on GWAS and two-step Mendelian randomization (MR).

Mendelian randomization (MR) is a novel epidemiologic method that uses a genetic variation to infer a causal correlation between exposure and outcome based on genetic variation closely related to exposure as potentially unconstrained instrumental variables (IVs). First proposed by Katan in 1986 to disclose whether low LDL cholesterol levels increase cancer risk, MR has become increasingly popular as genetic information on health and disease has expanded with data from genome-wide association studies and genome sequencing (17). The cardinal principle of MR assumes that genetic variants are randomly allocated at conception, mimicking the randomized controlled studies and operating independently of potential confounding variables such as environmental and lifestyle factors. MR also avoids the bias from reverse causality because diseases cannot affect genotypes. It provides a way to answer questions of causality without the typical errors that affect conclusions prevalent in many traditional epidemiological methods (18, 19). Based on the fact that the prevalence of obesity and long-COVID and the threat of air pollution have not yet been fully controlled, in order to reduce the morbidity and mortality of COVID-19, to better detect and prevent the occurrence of related diseases in key populations, and to advocate the importance of environmental protection, we discussed in detail the relationship between the three. In this paper, we applied an initial MR to explore the causal role of air pollution on COVID-19 and then explored whether obesity plays an intermediary role using a two-step MR. In step one, genetic IVs robustly associated with air pollution (PM2.5, NOx, NO_2_) were used to assess the causal relationship with obesity. In step two, genetic IVs robustly associated with obesity were used to assess the causal relationship with COVID-19 risk (susceptibility, hospitalization, severity).

## Methods

### Data sources for air pollution

All data used for analysis in our paper were obtained from publicly available GWAS datasets and therefore do not require ethical approval or informed consent. Summary statistics of GWAS data for the participants exposed in different levels of air pollution (PM2.5, NOx, NO_2_) were obtained from the UK Biobank (UKB) (20). The UK Biobank is a large biomedical database and research resource, an organization that collected in-depth genetic and health information on approximately 500,000 UK participants between 2006 and 2010 through questionnaires, medical tests and other methods. New data are added regularly to the database, which is accessible to all researchers worldwide. The extents of air pollution were estimated in different sites in the UK by a land use regression for annual average 2010 (21). The mean PM2.5 was 9.99 ± 1.06 micro-g/m^3^, ranging from 8.17 - 21.31 micro-g/m^3^, in a GWAS including 423,796 individuals and a total of 9,851,867 single-nucleotide polymorphisms (SNPs) (22). The mean NO_2_ was 26.71 ± 7.58 micro-g/m^3^, ranging from 12.93 - 108.49 micro-g/m^3^, in a GWAS including 456,380 individuals and a total of 9,851,867 SNPs (23). The mean NOx was 44.11 ± 15.53 micro-g/m^3^, ranging from 19.74 - 265.94 micro-g/m^3^, in a GWAS including 456,380 individuals and a total of 9,851,867 SNPs (23).

### Data sources for obesity

Summary statistics of obesity were obtained from the GIANT consortium (https://portals.broadinstitute.org/collaboration/giant/index.php/GIANT_consortium_data_files) (24, 25) and Liu et al. GWAS meta-analyses (26). The GIANT Alliance is an international collaboration of researchers from different groups, institutions, countries and research organizations. The consortium aims to identify genetic loci that regulate human size and shape (including obesity-related traits such as height, BMI, waist circumference, etc.), primarily through meta-analysis of genome-wide association data and other large-scale genetic datasets. The GWAS of the volume of abdominal subcutaneous adipose tissue (ASAT) and visceral adipose tissue (VAT) included 32,860 individuals and a total of 9,275,407 SNPs, respectively. The GWAS of body mass index (BMI) included 681,275 individuals and a total of 2,336,260 SNPs. The GWAS of hip circumference (HC) included 213,038 individuals and a total of 2,559,739 SNPs. The GWAS of obesity class 1 (OB1) included 98,697 individuals and a total of 2,380,428 SNPs. The GWAS of obesity class 2 (OB2) included 72,546 individuals and a total of 2,331,456 SNPs. The GWAS of obesity class 3 (OB3) included 50,364 individuals and a total of 2,250,779 SNPs. The GWAS of waist circumference (WC) included 232,101 individuals and a total of 2,565,408 SNPs. The GWAS of waist-to-hip ratio (WHR) included 212,244 individuals and a total of 2,560,782 SNPs.

### Data sources for COVID-19

Summary statistics of COVID-19 were obtained from the COVID-19 host genetic websites released on April 8, 2022 (round 7, GRCh38, https://www.covid19hg.org/results/r7/) (27). The GWAS of COVID-19 susceptibility included 2,597,856 cases and 14,496,978 SNPs (C2_ALL_eur_leave_23andme). The GWAS of COVID-19 hospitalization included 2,095,324 cases and 12,469,431 SNPs (B2_ALL_eur_leave_23andme). The GWAS of COVID-19 severity included 1,086,211 cases and 12,174,527 SNPs (A2_ALL_eur_leave_23andme).

### Mendelian randomization

Three principles genetic tools were followed for MR analysis: a. genetic tools were strongly correlated with corresponding exposures (*p* < 5×10^-5^), which could avoid the possibility of insufficient powered instrumental variables (IVs) and has been applied on previous studies (28, 29); b. genetic tools were independent of outcomes and could only influence outcome through exposure; and c. when conducting MRs between air pollutions and COVID-19 risks, the genetic tools were independent of the mediators (30). The IVs of SNPs were conjugated using the PLINK algorithm (LD < 0.001 and < 10 MB from the index variant) to select independent IVs. The F-statistic was calculated by the (R^2^/K)/[(1-R^2^) (N-K-1)], where K is the number of SNP, N is the sample size, R^2^ is the variance explained by SNPs calculated by 2*EAF*(1-EAF) * (Beta/SE)^2^. The IVs with F < 10 were excluded to retain the reliable SNPs which robustly represented the exposures. The random effects inverse variance weighting (IVW) was used as the main analysis method, which combines the Wald ratios of the causal effect of each SNP on the outcome and provides the most accurate estimates (31). Meanwhile, MR-Egger regression method and weighted median method were used as supplements to IVW. Moreover, MR-Egger intercept test, Cochran’s Q test, MR-Egger intercept test and leave-one-out analysis were used to determine the presence of pleiotropy and to assess the reliability of the results.

### Mediated effects analysis

Three beta values would be gained through two-step MR, namely beta0 (initial MR of exposures on outcomes), beta1 (step one MR of exposures on mediators), and beta2 (step two MR of mediators on outcomes). The results are interpreted as follows: 1. If beta0, beta1 and beta2 are all significant, this indicates that there is a causal association from exposure to outcome and that this association may be partially mediated by the mediating variable; 2. If beta0 is not significant but both beta1 and beta2 are significant, meanwhile the quantified indirect effects are significant, this indicates that the causal association from exposure to outcome is indirect and mediated by this variable; 3. If beta0 is significant, at least one of beta1 and beta2 is insignificant, indicating that there is no mediating effect mediated by this mediating variable in the causal association from exposure to outcome (32).

The indirect effects were recognized as the effects of exposures on outcomes mediated through the causal mediators, which was quantified by the product of coefficients method (32, 33).

### Statistical analysis

Results of Mendelian analysis were presented using beta, 95% confidence interval (95%CI) and *p* values. *P* < 0.05 was considered as statistical significance. R (version 4.0.5) packages (TwoSampleMR, version 0.5.6) was applied to perform statistical analysis.

## Results

**Figure 1:**
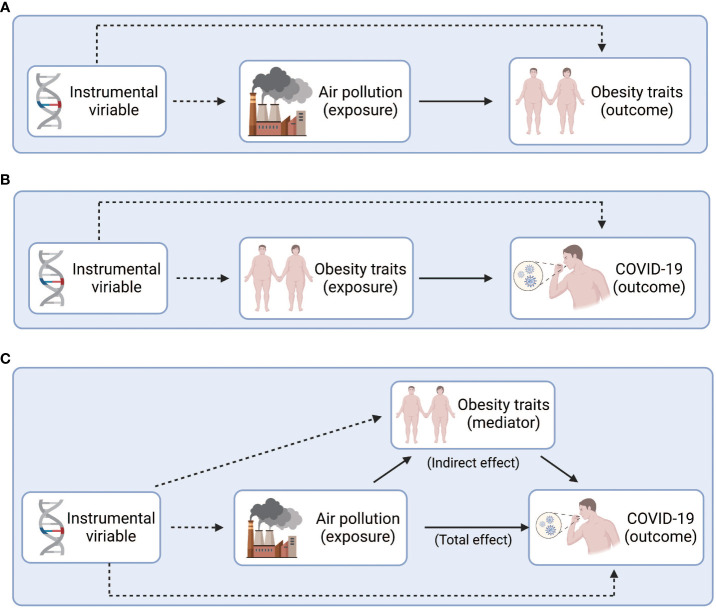
Study design overview. **(A)** Explore the causal relationship between air pollution and obesity. **(B)** Explore the causal relationship between obesity and COVID-19. **(C)** Explore the intermediary role of obesity between air pollution and COVID-19. Figure built by the Biorender.

**Figure 2:**
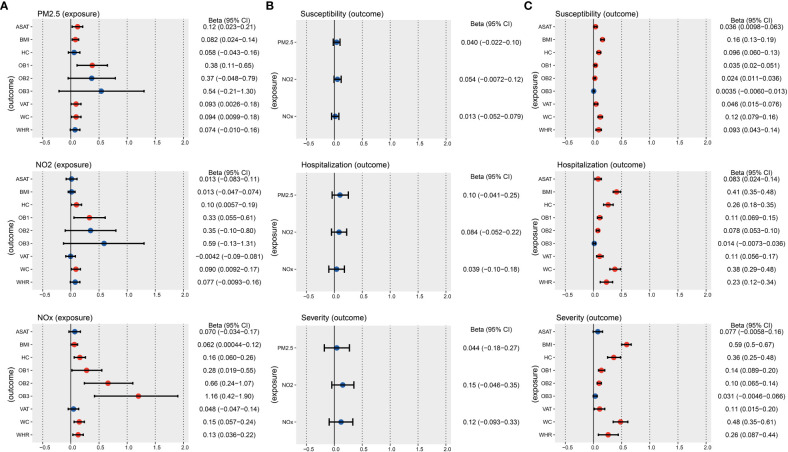
IVW results of the causal relationship between air pollution, obesity, and COVID-19 risk. **(A)** IVW results of the causal relationship between air pollution and obesity. **(B)** IVW results of the causal relationship between air pollution and COVID-19. **(C)** IVW results of the causal relationship between obesity and COVID-19. The red color means the p-value is less than 0.05.

We used graphical figures to demonstrate the entire analytical process of Mendelian randomization (**Figure 1**). In summary, 251, 295 and 254 index SNPs were obtained to demonstrate the genetic characteristics of PM2.5, NO_2_, and NOx, respectively (**Supplementary Tables S2**–**4**); 155, 837, 178, 96, 77, 38, 152, 165, 136 index SNPs were obtained to demonstrate the genetic characteristics of ASAT, BMI, HC, OB1, OB2, OB3, VAT, WC, and WHR, respectively (**Supplementary Tables S5**-**13**). First, we performed two sample MR to calculated the casual relationship between air pollution and obesity traits (**Figure 2A** and **Table 1**). IVW analysis indicated a positive causal relationship between PM2.5 exposure and ASAT (*p* = 1.49E-02), BMI (*p* = 5.73E-03), OB1 (*p* = 6.31E-03), VAT (*p* = 4.38E-02), WC (*p* = 2.85E-02); a positive causal relationship between NO_2_ exposure and HC (*p* = 3.77E-02), OB1 (*p* = 1.90E-02), WC (*p* = 2.90E-02); a positive causal relationship between NOx exposure and BMI (*p* = 4.84E-02), HC (*p* = 1.74E-03), OB1 (*p* = 3.60E-02), OB2 (*p* = 1.85E-03), OB3 (*p* = 2.10E-03), WC (*p* = 1.61E-03), WHR (*p* = 6.37E-03).

**Table 1:** MR results of air pollution effects on obesity traits by IVW.

Exposure	Outcome	nSNP	Beta	LCI	UCI	p
PM2.5	ASAT	240	0.12	0.023	0.21	**1.49E-02**
	BMI	99	0.082	0.024	0.14	**5.73E-03**
	HC	119	0.058	-0.043	0.16	2.63E-01
	OB1	113	0.38	0.11	0.65	**6.31E-03**
	OB2	113	0.37	-0.048	0.79	8.27E-02
	OB3	113	0.54	-0.21	1.30	1.59E-01
	VAT	240	0.093	0.0026	0.18	**4.38E-02**
	WC	118	0.094	0.0099	0.18	**2.85E-02**
	WHR	119	0.074	-0.010	0.16	8.44E-02
NO_2_	ASAT	283	0.013	-0.083	0.11	7.87E-01
	BMI	110	0.013	-0.047	0.074	6.69E-01
	HC	133	0.10	0.0057	0.19	**3.77E-02**
	OB1	127	0.33	0.055	0.61	**1.90E-02**
	OB2	127	0.35	-0.10	0.80	1.27E-01
	OB3	123	0.59	-0.13	1.31	1.10E-01
	VAT	283	-0.0042	-0.090	0.081	9.23E-01
	WC	131	0.090	0.0092	0.17	**2.90E-02**
	WHR	132	0.077	-0.0093	0.16	8.04E-02
NOx	ASAT	240	0.070	-0.034	0.17	1.87E-01
	BMI	99	0.062	0.00044	0.12	**4.84E-02**
	HC	119	0.16	0.060	0.26	**1.74E-03**
	OB1	113	0.28	0.019	0.55	**3.60E-02**
	OB2	113	0.66	0.24	1.07	**1.85E-03**
	OB3	113	1.16	0.42	1.90	**2.10E-03**
	VAT	240	0.048	-0.047	0.14	3.20E-01
	WC	118	0.15	0.057	0.24	**1.61E-03**
	WHR	119	0.13	0.036	0.22	**6.37E-03**

Beta = log (OR). P < 0.05 were bolded.

PM2.5, Particulate matter air pollution; NO_2_, Nitrogen dioxide; NO_X_, Nitrogen oxides; ASAT, abdominal subcutaneous adipose tissue; BMI, body mass index; HC, hip circumference; OB1, obesity class 1; OB2, obesity class 2; OB3, obesity class 3; VAT, visceral adipose tissue; WC, waist circumference; WHR, waist-to-hip ratio; UCI, Upper confidence interval; LCI, Lower confidence interval.

Second, we performed two sample MR to calculated the casual relationship between air pollution and COVID-19 (**Figure 2B** and **Table 2**). IVW analysis suggested that there is no direct causal relationship between them.

**Table 2:** MR results of air pollution effects on COVID-19 by IVW.

Exposure	Outcome	nSNP	Beta	LCI	UCI	p
PM2.5	Susceptibility	249	0.040	-0.022	0.10	2.06E-01
NO2		289	0.054	-0.0072	0.12	8.35E-02
NOx		247	0.013	-0.052	0.079	6.92E-01
PM2.5	Hospitalization	247	0.10	-0.041	0.25	1.59E-01
NO2		287	0.084	-0.052	0.22	2.24E-01
NOx		247	0.039	-0.10	0.18	5.90E-01
PM2.5	Severity	247	0.044	-0.18	0.27	6.97E-01
NO2		288	0.15	-0.046	0.35	1.31E-01
NOx		247	0.12	-0.093	0.33	2.70E-01

Beta = log (OR).

PM2.5, Particulate matter air pollution; NO2, Nitrogen dioxide; NO_X_, Nitrogen oxides; UCI, Upper confidence interval; LCI, Lower confidence interval.

Third, we performed two sample MR to calculated the casual correlation between obesity traits and COVID-19 (**Figure 2C** and **Table 3**). IVW analysis indicated a positive causal relationship between ASAT and COVID-19 susceptibility (*p* = 7.35E-03), COVID-19 hospitalization (*p* = 5.88E-03). IVW analysis indicated a positive causal relationship between BMI and COVID-19 susceptibility (*p* = 1.74E-27), COVID-19 hospitalization (*p* = 2.46E-40), COVID-19 severity (*p* = 1.44E-40). IVW analysis indicated a positive causal relationship between HC and COVID-19 susceptibility (*p* = 1.95E-07), COVID-19 hospitalization (*p* = 1.09E-09), COVID-19 severity (*p* = 1.64E-09). We also found a positive causal relationship between OB1 and COVID-19 susceptibility (*p* = 1.20E-05), COVID-19 hospitalization (*p* = 7.97E-08), COVID-19 severity (*p* = 1.75E-07). There was a positive causal relationship between OB2 and COVID-19 susceptibility (*p* = 1.51E-04), COVID-19 hospitalization (*p* = 5.77E-10), COVID-19 severity (*p* = 1.20E-07).

**Table 3:** MR results of obesity trait effects on COVID-19 by IVW.

Exposure	Outcome	nSNP	Beta	LCI	UCI	p
ASAT	Susceptibility	146	0.036	0.0098	0.063	**7.35E-03**
BMI		812	0.16	0.13	0.19	**1.74E-27**
HC		174	0.096	0.060	0.13	**1.95E-07**
OB1		88	0.035	0.020	0.051	**1.20E-05**
OB2		75	0.024	0.011	0.036	**1.51E-04**
OB3		37	0.0035	-0.0060	0.013	4.75E-01
VAT		140	0.046	0.015	0.076	**3.33E-03**
WC		160	0.12	0.079	0.16	**1.44E-08**
WHR		130	0.093	0.043	0.14	**3.03E-04**
ASAT	Hospitalization	140	0.083	0.024	0.14	**5.88E-03**
BMI		806	0.41	0.35	0.48	**2.46E-40**
HC		174	0.26	0.18	0.35	**1.09E-09**
OB1		88	0.11	0.069	0.15	**7.97E-08**
OB2		74	0.078	0.053	0.10	**5.77E-10**
OB3		37	0.014	-0.0073	0.036	1.95E-01
VAT		134	0.11	0.056	0.17	**1.23E-04**
WC		160	0.38	0.29	0.48	**3.23E-16**
WHR		130	0.23	0.12	0.34	**4.37E-05**
ASAT	Severity	141	0.077	-0.0058	0.16	6.84E-02
BMI		811	0.59	0.50	0.67	**1.44E-40**
HC		174	0.36	0.25	0.48	**1.64E-09**
OB1		89	0.14	0.089	0.20	**1.75E-07**
OB2		75	0.10	0.065	0.14	**1.20E-07**
OB3		37	0.031	-0.0046	0.066	8.87E-02
VAT		134	0.11	0.015	0.20	**2.24E-02**
WC		160	0.48	0.35	0.61	**5.99E-13**
WHR		131	0.26	0.087	0.44	**3.32E-03**

Beta = log (OR). P < 0.05 were bolded.

Abdominal subcutaneous adipose tissue, ASAT; Body mass index, BMI; Hip circumference, HC; OB1, obesity class 1; OB2, obesity class 2; OB3, obesity class 3; Visceral adipose tissue, VAT; Waist circumference, WC; Waist-to-hip ratio, WHR; Upper confidence interval, UCI, Lower confidence interval, LCI.

Meanwhile, our paper revealed a positive causal relationship between VAT and COVID-19 susceptibility (*p* = 3.33E-03), COVID-19 hospitalization (*p* = 1.23E-04), COVID-19 severity (*p* = 2.24E-2). WC was positively related to the risks of COVID-19 susceptibility (*p* = 1.44E-08), COVID-19 hospitalization (*p* = 3.23E-16), COVID-19 severity (*p* = 5.99E-13). WHR was positively related to the risks of COVID-19 susceptibility (*p* = 3.03E-04), COVID-19 hospitalization (*p* = 4.37E-05), COVID-19 severity (*p* = 3.32E-03). In addition, MR Egger and Weighted median were used as supplementary analysis methods for IVW, and detailed results are presented in **Supplementary Table S14**. The above results suggested that air pollution may indirectly increase the risk of COVID-19 by affecting obesity, with obesity traits playing a mediating role.

**Figure 3:**
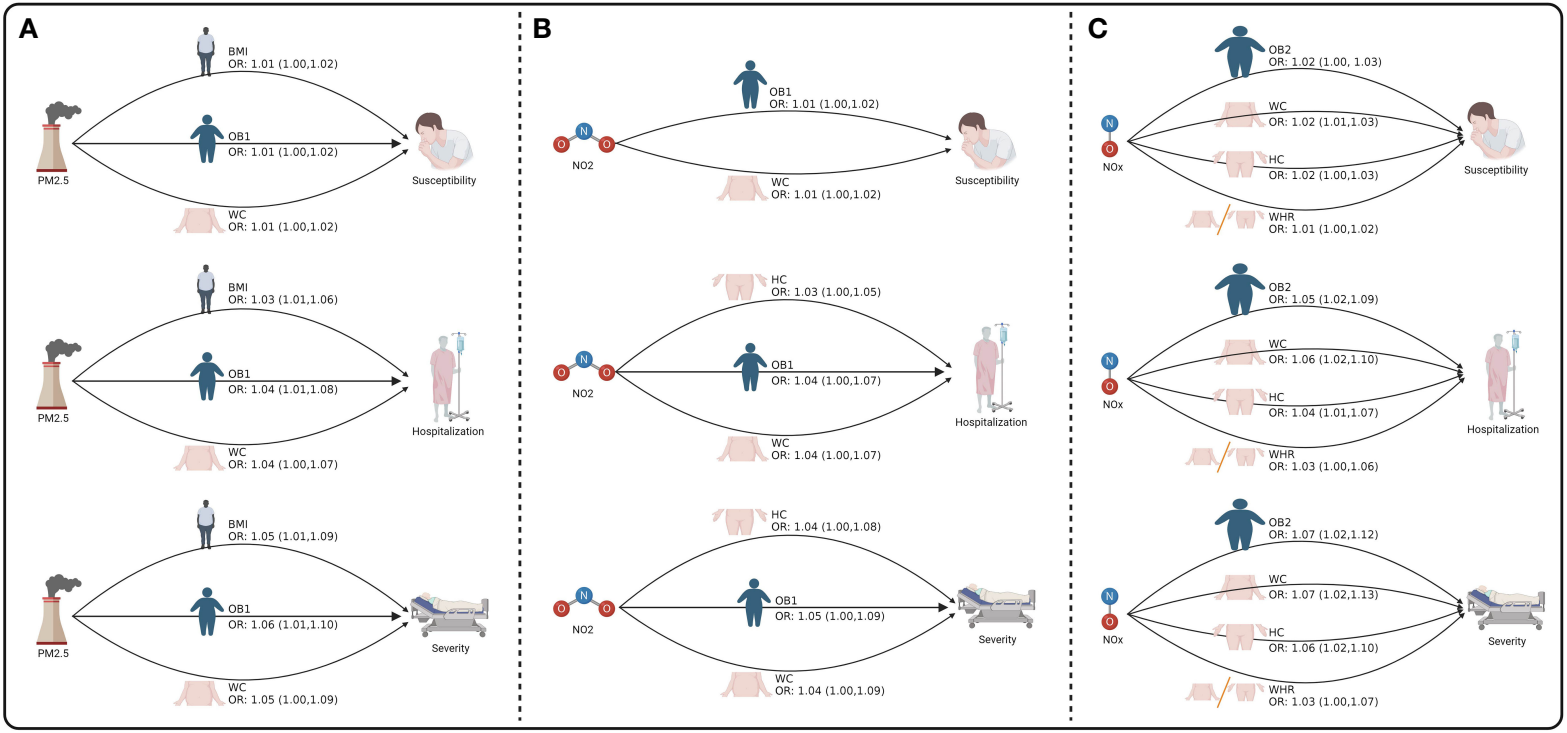
The mediating role of obesity between air pollution and COVID-19. **(A)** The mediating role of obesity between PM2.5 and COVID-19. **(B)** The mediating role of obesity between NO2 and COVID-19. **(C)** The mediating role of obesity between NOX and COVID-19. Figure built by the Biorender.

Next, we to calculate the indirect effect played by obesity traits in air pollution affecting COVID-19 (**Figure 3** and **Supplementary Table S16**). Our results found that PM2.5 indirectly increased the risk of COVID-19 susceptibility by affecting BMI (OR = 1.01, 95% CI = 1.00-1.02, *p* = 7.41E-03), OB1 (OR = 1.01, 95% CI = 1.00-1.02, *p* = 2.05E-02), and WC (OR = 1.01, 95% CI = 1.00-1.02, *p* = 4.10E-02). PM2.5 also indirectly increased the risk of COVID-19 hospitalization by affecting BMI (OR = 1.03, 95% CI = 1.01-1.06, *p* = 6.82E-03), OB1 (OR = 1.04, 95% CI = 1.01-1.08, *p* = 1.49E-02), and WC (OR = 1.04, 95% CI = 1.00-1.07, *p* = 3.44E-02). Moreover, PM2.5 indirectly increased the risk of COVID-19 severity by affecting BMI (OR = 1.05, 95% CI = 1.01-1.09, *p* = 6.82E-03), OB1 (OR = 1.06, 95% CI = 1.01-1.10, *p* = 1.55E-02), and WC (OR = 1.05, 95% CI = 1.00-1.09, *p* = 3.61E-02) (**Figure 3A** and **Table 4**). Moreover, NO_2_ indirectly increased the risk of COVID-19 susceptibility by affecting OB1 (OR = 1.01, 95% CI = 1.00-1.02, *p* = 3.87E-02) and WC (OR = 1.01, 95% CI = 1.00-1.02, *p* = 4.16E-02). Our data also indicated that NO_2_ indirectly increased the risk of COVID-19 hospitalization by affecting HC (OR = 1.03, 95% CI = 1.00-1.05, *p* = 4.92E-02), OB1 (OR = 1.04, 95% CI = 1.00-1.07, *p* = 3.16E-02), and WC (OR = 1.04, 95% CI = 1.00-1.07, *p* = 3.49E-02) (**Figure 3B** and **Table 4**).

**Table 4:** Significant indirect effects of air pollution on COVID-19 mediated by obesity traits.

Exposure	Mediate	Outcome	OR	LCI	UCI	p
PM2.5	BMI	Susceptibility	1.01	1.00	1.02	7.41E-03
	OB1		1.01	1.00	1.02	2.05E-02
	WC		1.01	1.00	1.02	4.10E-02
	BMI	Hospitalization	1.03	1.01	1.06	6.82E-03
	OB1		1.04	1.01	1.08	1.49E-02
	WC		1.04	1.00	1.07	3.44E-02
	BMI	Severity	1.05	1.01	1.09	6.82E-03
	OB1		1.06	1.01	1.10	1.55E-02
	WC		1.05	1.00	1.09	3.61E-02
NO2	OB1	Susceptibility	1.01	1.00	1.02	3.87E-02
	WC		1.01	1.00	1.02	4.16E-02
	HC	Hospitalization	1.03	1.00	1.05	4.92E-02
	OB1		1.04	1.00	1.07	3.16E-02
	WC		1.04	1.00	1.07	3.49E-02
	HC	Severity	1.04	1.00	1.08	4.95E-02
	OB1		1.05	1.00	1.09	3.24E-02
	WC		1.04	1.00	1.09	3.67E-02
NOx	HC	Susceptibility	1.02	1.00	1.03	7.29E-03
	OB2		1.02	1.00	1.03	1.62E-02
	WC		1.02	1.01	1.03	5.85E-03
	WHR		1.01	1.00	1.02	2.95E-02
	HC	Hospitalization	1.04	1.01	1.07	5.35E-03
	OB2		1.05	1.02	1.09	5.41E-03
	WC		1.06	1.02	1.10	3.26E-03
	WHR		1.03	1.00	1.06	2.33E-02
	HC	Severity	1.06	1.02	1.10	5.45E-03
	OB2		1.07	1.02	1.12	7.30E-03
	WC		1.07	1.02	1.13	3.86E-03
	WHR		1.03	1.00	1.07	4.56E-02

Beta = log (OR).

PM2.5, Particulate matter air pollution; NO_2_, Nitrogen dioxide; NO_X_, Nitrogen oxides; ASAT, abdominal subcutaneous adipose tissue; BMI, body mass index; HC, hip circumference; OB1, obesity class 1; OB2, obesity class 2; OB3, obesity class 3; VAT, visceral adipose tissue; WC, waist circumference; WHR, waist-to-hip ratio; UCI, Upper confidence interval; LCI, Lower confidence interval.

Furthermore, NOx indirectly increased the risk of COVID-19 susceptibility by affecting HC (OR = 1.02, 95% CI = 1.00-1.03, *p* = 7.29E-03), OB2 (OR = 1.02, 95% CI = 1.00-1.03, *p* = 1.62E-03), WC (OR = 1.02, 95% CI = 1.01-1.03, *p* = 5.85E-03), and WHR (OR = 1.01, 95% CI = 1.00-1.02, *p* = 2.95E-02). NOx indirectly increased the risk of COVID-19 hospitalization by affecting HC (OR = 1.04, 95% CI = 1.01-1.07, *p* = 5.35E-03), OB2 (OR = 1.05, 95% CI = 1.02-1.09, *p* = 5.41E-03), WC (OR = 1.06, 95% CI = 1.02-1.10, *p* = 3.26E-03), and WHR (OR = 1.03, 95% CI = 1.00-1.06, *p* = 2.33E-02). NOx indirectly increased the risk of COVID-19 severity by affecting HC (OR = 1.06, 95% CI = 1.02-1.10, *p* = 5.45E-03), OB2 (OR = 1.07, 95% CI = 1.02-1.12, *p* = 7.30E-03), WC (OR = 1.07, 95% CI = 1.02-1.13, *p* = 3.86E-03), and WHR (OR = 1.03, 95% CI = 1.00-1.07, *p* = 4.56E-02) (**Figure 3C** and **Table 4**).

Finally, to enhance the reliability of our results, we used MR-Egger-intercept test, Cochran’s Q test, and leave-one-out analysis to perform sensitivity analysis on our results (**Supplementary Figures S1**–**3** and **Supplementary Table S15**). The results of Cochran’s Q test in IVW showed that there is basically no heterogeneity, and the MR-Egger-intercept test and leave-one-out analysis showed that our results are quite reliable. The F-statistic for the instrumental variables were all greater than 10, also indicating the reliability of the results.

## Discussion

To date, several epidemiological studies have found that certain airborne pollutants are risk factors for obesity and COVID-19 (34), but the limitations of traditional observational study methods make it difficult to establish a causal relationship between them. In this paper, we conducted two-sample and two-step MR to assess the role of air pollution exposure on obesity traits and COVID-19 based on large-scale GWAS datasets. We found that prolonged exposure to three air pollutant molecules (PM2.5, NO_2_, and NOx) increased the risk of obesity, suggesting a causal relationship between them. There is also a causal relationship between obesity traits and COVID-19 susceptibility, hospitalization and severity. Chronic exposure to three air pollution molecules (PM2.5, NO_2_, and NOx) did not directly contribute to COVID-19 risk, but rather increased COVID-19 susceptibility, hospitalization and severity by affecting obesity. Given these findings, we believe that among those living in areas with heavy air pollution, maintaining a healthy weight may help prevent COVID-19 infections.

In recent years, numerous epidemiological studies have explored the relationship between long-term exposure to air pollution and obesity in different regions and populations. However, the findings of these observational studies are controversial. To date, most of the current evidence supports that air pollution can contribute to the development of obesity in children and adults, but there is also a small amount of evidence suggesting no relationship or a negative association between the two. For example, Qian Guo et al. (35) found that the risk of childhood obesity elevated by 10.0% (95% CI = 3.0-16.0%) for each 10 μg/m^3^ increment in PM2.5 exposure. Meanwhile, the risk associated with PM2.5 was significantly higher in groups that were older or lived in urban areas. Another prospective cohort study suggested a negative correlation between decreasing PM2.5 concentrations and the prevalence of obesity in children and adolescents, suggesting that cleaning up airborne pollutants could prevent the development of obesity in these populations (36). Sara Fioravanti et al. (37) found no association between exposure to vehicle traffic-related air pollutants and obesity-related indicators such as BMI and abdominal fat during childhood. Jian V Huang et al. (38) found that high air pollutants in childhood were related to a lower BMI at age 13 to 15 years.

Limited reports suggested that air pollution may contribute to obesity by affecting adipocyte function through mechanisms such as cellular inflammation or oxidative stress. For example, animal experiments have shown that PM2.5 exposure may cause metabolic disorders of lipid synthases and fatty acid transporter proteins in adipose tissue and liver through the Nrf2/PPAR pathway, leading to adipose tissue overgrowth (39). Cellular experiments have shown that acute or chronic exposure to PM2.5 can lead to the overproduction of cytoplasmic reactive oxygen species (ROS), induce oxidative damage and activate the oxygen-sensitive NRF2 and NF-kB signaling pathways (40). In current study, we confirmed, using Mendelian randomization analysis, PM2.5 as a direct cause of various obesity-related parameters such as ASAT, BMI, OB1, VAT and WC. In addition, NO_2_ is a direct cause of elevated risk for HC, OB1 and WC; and NOx is a direct cause of elevated risk for BMI, HC, OB1, OB2, OB3, WC and WHR.

The prevalence of overweight/obesity has continued to increase worldwide over the past half century, currently affecting 2 billion adults, with 770 million having obesity (41). Obesity is a major health challenge because it greatly increases the risk of many chronic diseases, which leads to reduced quality of life and life expectancy (42). In particular, with the focus on COVID-19 from 2019, more and more people are focusing on the correlation between obesity and COVID-19. Studies have shown that obesity may influence the response and prognosis of COVID-19 through a variety of mechanisms such as immune response, metabolic abnormalities and the gut-lung axis (43). An observational study that included 5,279 participants showed that COVID-19 patients with a BMI ≥40 kg/m^2^ had a more than 2-fold increased risk of hospitalization compared with patients of normal weight (OR = 2. 5; 95% CI = 1.8-3.4), after excluding the effects of age, gender, and race (44). Similarly, in another study conducted by Norbert Stefan et al. (45), the adjusted OR for admission of COVID-19 inpatients with currently states obesity or BMI >= 30 kg/m^2^ in the past 12 months was 1.43. Meanwhile, many evidence support that obesity is a key indicator for the severity of COVID-19 inpatients (46, 47). These studies generally concluded that obesity prolongs the time to intensive care unit admission, intubation, and mechanical ventilation in COVID-19 patients (48).

Another observational study including more than 140,000 COVID-19 patients showed that the adjusted risk ratio for patients with BMI >=45 kg/m^2^ admitted to intensive care unit (ICU) was 1.16 (95% CI = 1.11-1.20). And, the adjusted risk ratio for patients on mechanical ventilation increased from 1.12 (25-29.9 kg/m^2^) to 2.08 (BMI >= 45 kg/m^2^) (49). Further studies suggested that obesity may increase the susceptibility and severity of COVID-19 by upregulating the expression of angiotensin-converting enzyme 2 receptors that bind to SARS-CoV-2 (50). Recent studies suggested that obesity may potentially reduce the long-term efficacy of COVID-19 vaccine by affecting the collective immune system, suggesting that we should closely monitor the efficacy of COVID-19 vaccination in this vulnerable group of obesity (51). It has been shown that the adipocyte membrane receptors ACE2, DPP4 and CD147 as well as the expression of SARS-CoV-2 entry protease-furin are upregulated in patients with obesity (52). These receptors and proteins may therefore be potential targets for SARS-CoV-2 attack and contribute to the severe consequences of COVID-19 in patients with obesity by enhancing systemic inflammation and immune responses. However, these observational studies do not provide powerful evidence for a causal correlation between obesity and COVID-19 risk. In our MR study, we found that most obesity traits, including BMI, HC, OB1, OB2, VAT, WC and WHR, directly increase COVID-19 risk. Our findings are generally consistent with previous observational studies and will provide theoretical support for future prevention of COVID-19 and improved prognosis of patients with COVID-19.

Long-term exposure to air pollution can damage the body’s immune system to defend against external pathogens, which can cause a lot of diseases. In recent years, several studies have shown that exposure to air pollution, such as PM2.5, NO_2_, and O_3_, can increase the susceptibility and severity of COVID-19 (9). Although the molecular mechanisms by which pollutant exposure affects the pathogenesis of COVID-19 remain unknown. Studies suggested that air pollutants may contribute to virus transmission by modulating mucociliary clearance, altered proteases required for viruses, interferon production, mediated autophagy, immune presenting cell activation, and epithelial cell permeability (34). An epidemiological study from the United Kingdom found that PM2.5 was a major contributor to COVID-19 hospitalization in England, with a 12% increase in COVID-19 cases for every 1 cubic meter increase in the long-term mean PM2.5 (53). Moreover, the relationship between air pollution and COVID-19 mortality remained significant after adjusting for other relevant variables.

An observational study that collected data on COVD-19 from 3,087 countries in the United States showed that a 1 µg/m^3^ elevation in PM2.5 increased COVID-19 mortality by 8% (95% CI: 2%-15%) (54). In this present study, we used Mendelian randomization and found no evidence that air pollution directly increased COVID-19 risk. Interestingly, we found that air pollution can indirectly increase hospitalization, susceptibility and severity of COVID-19 by contributing to obesity. We found that PM2.5 and NOx increased COVID-19 risk (hospitalization and susceptibility) through BMI. PM2.5, NO2 and NOx increased COVID-19 susceptibility through WC. NO_2_ and NOx increased COVID-19 hospitalization through HC. NOx increased COVID-19 hospitalization through WC and WHR, and increased COVID-19 severity through WC and HC. More attention should be paid to those with obesity living in heavy air pollution in terms of COVID-19 prevention and protection, because obesity caused by air pollution might mediate increasing COVID-19 susceptibility, hospitalization and severity. Encouraging weight loss for this population is needed.

According to our understanding, this is the first systematic exploration of the causal correlation between air pollution and COVID-19 and whether obesity traits play a possible mediating role between them, using an MR approach. We used latest and comprehensive GWAS data (exposure, mediators, and outcomes) to systematically explore the relationship between the three, and will contribute in part to reducing the prevalence of obesity and COVID-19 in the future, as well as raising awareness of environmental protection. However, our study has several limitations. First, our studies were based on online public databases and, therefore, we could not validate them in our own or other databases. Second, obesity may be only one of many mediators of the risk of air pollution affecting COVID-19, and there may be other mediators between the two. Third, this paper only explored the causal correlation between air pollution, obesity and COVID-19 using Mendelian randomization, and the exact molecular mechanisms of the interactions still need to be explored in future studies.

## Conclusion

To summarize, this study exposes a causal relationship between air pollution, obesity and COVID-19. Our results suggested that air pollution can increase the risk of obesity and indirectly increase COVID-19 susceptibility and severity through mediating factors such as obesity. However, the specific mechanism of action between the three has not been clarified, and the detailed pathological mechanisms and molecular pathways need to be further explored in future studies.

## Ethics approval and consent to participate

All data used by this study were publicly available from participant studies with the approvement of the ethical standards committee related to human experimentation.

## Data availability statement

‘The original contributions presented in the study are included in the article/**Supplementary Materials**. Further inquiries can be directed to the corresponding authors.

## Author contributions

Writing -Original Draft, Methodology, Validation, Visualization: JW and JW-Z. Data Collection and Validation: JW and JW-Z. Conceptualization, Methodology, Supervision, Project Administration and Funding Acquisition: JW, JW-Z, XW, PL. All authors contributed to the article and approved the submitted version.
