# Supplementary figures and images for: The causal relationship between air pollution, obesity, and COVID-19 risk: a large-scale genetic correlation study
Source: Front Endocrinol (Lausanne). 2023 Oct 5;14:1221442. doi: 10.3389/fendo.2023.1221442 (PMC10585274; doi:10.3389/fendo.2023.1221442)

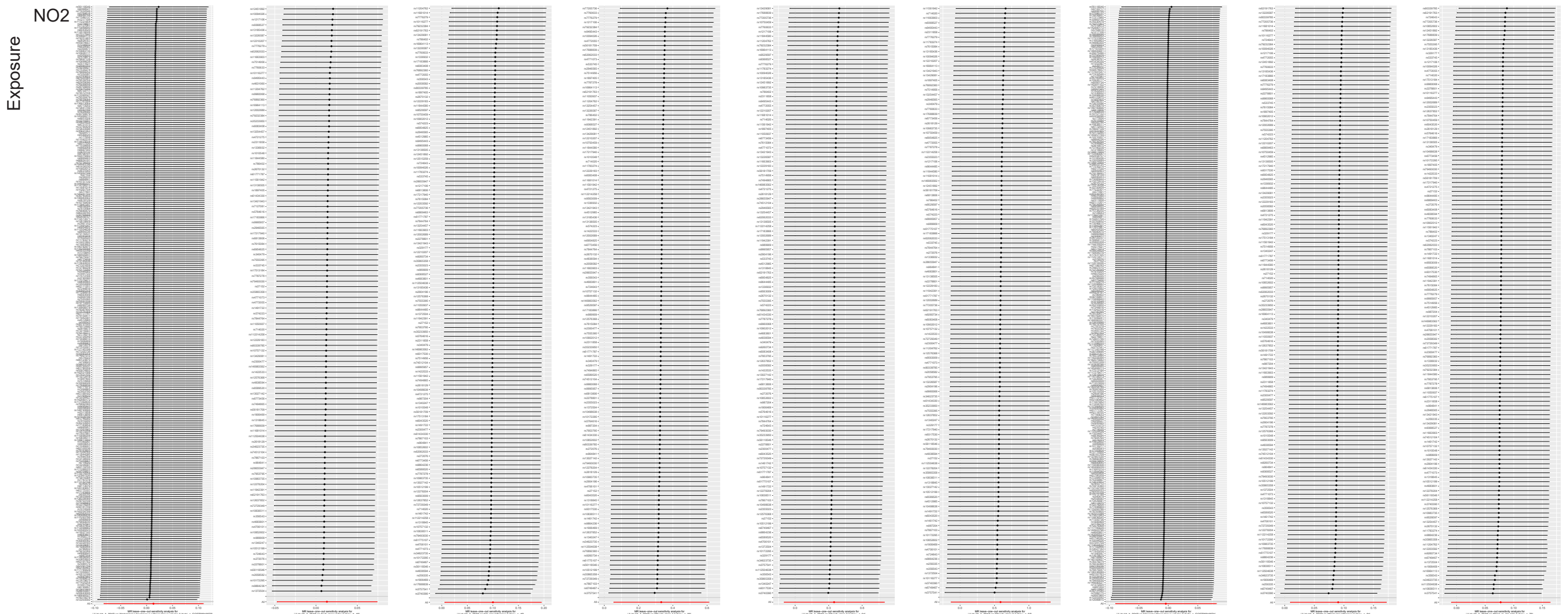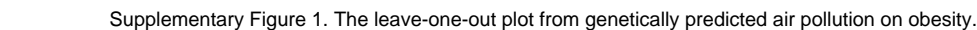

Supplement: Supplementary file 2 [file Image_1.pdf]

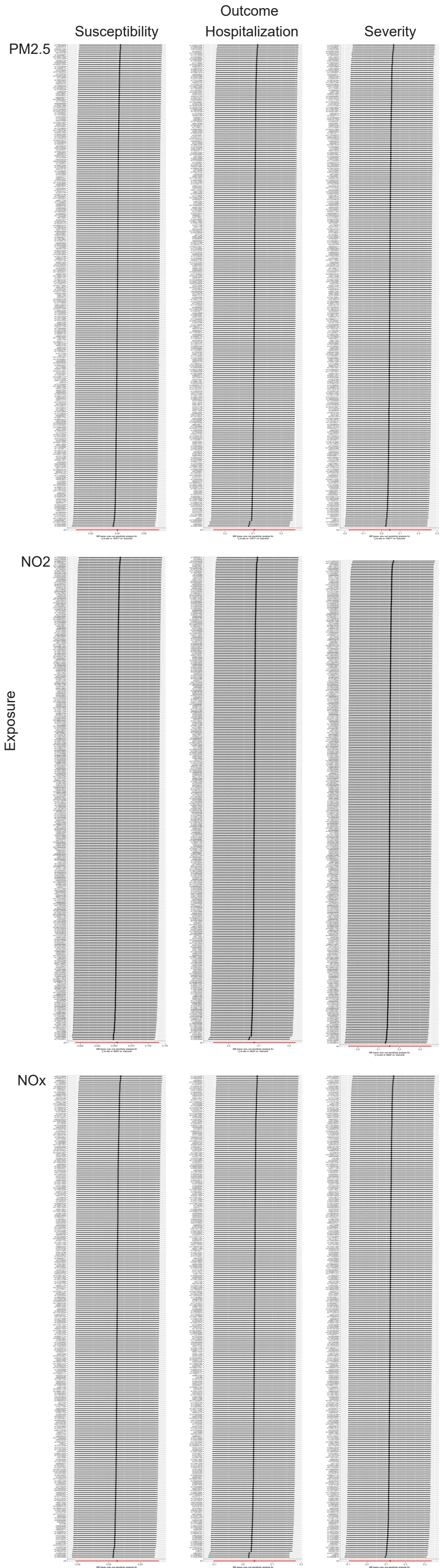

Supplement: Supplementary file 3 [file Image_2.pdf]

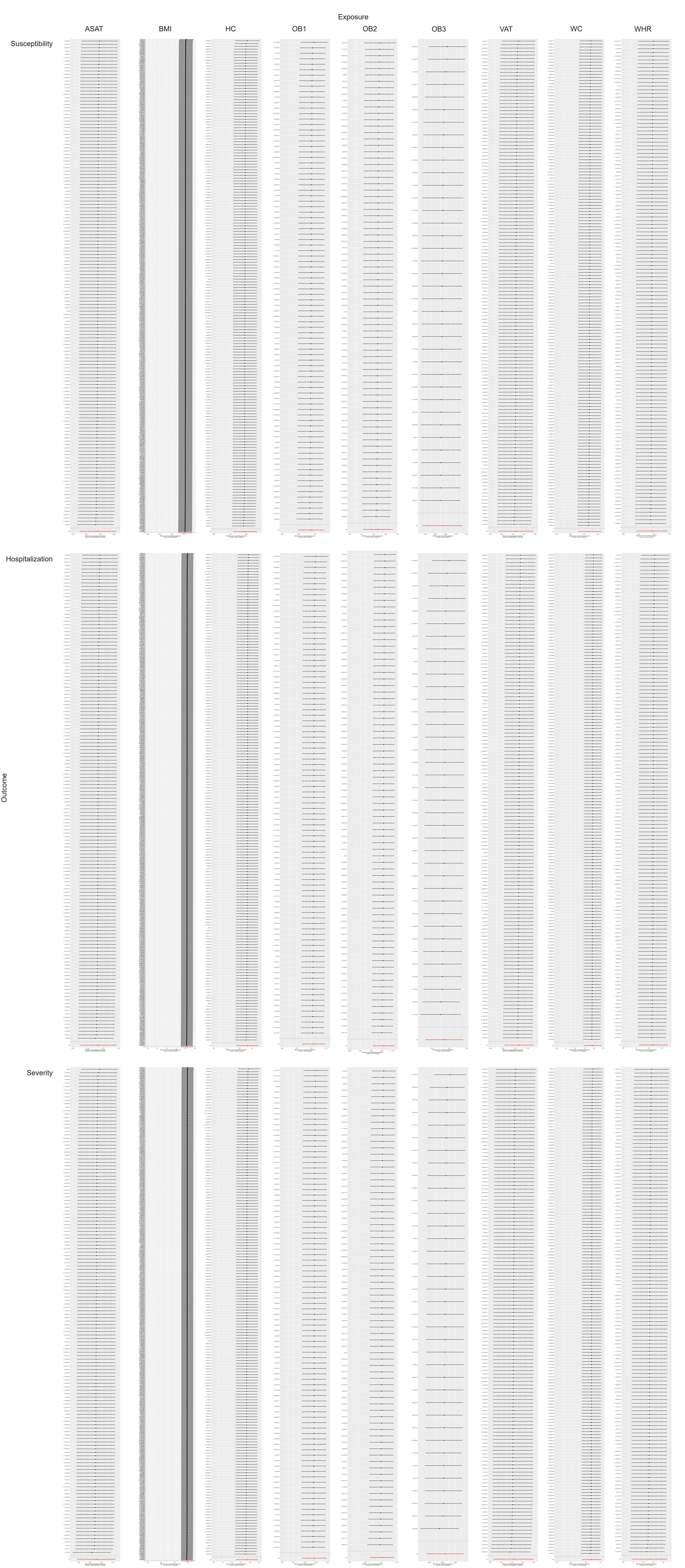

Supplement: Supplementary file 4 [file Image_3.pdf]
